# XPS valence band observable light-responsive system for photocatalytic acid Red114 dye decomposition using a ZnO–Cu_2_O heterojunction

**DOI:** 10.1016/j.heliyon.2024.e30802

**Published:** 2024-05-07

**Authors:** Nasrin Akter, Tanvir Ahmed, Imdadul Haque, Md Kamal Hossain, Gorungo Ray, Md Mufazzal Hossain, Md Sagirul Islam, Md Aftab Ali shaikh, Umme Sarmeen Akhtar

**Affiliations:** a Institute of Glass and Ceramic Research and Testing (IGCRT), Bangladesh Council of Scientific and Industrial Research (BCSIR), Dr. Qudrat-I-Khuda Road, Dhanmondi, Dhaka 1205, Bangladesh; b BCSIR Laboratories Dhaka, Bangladesh Council of Scientific and Industrial Research (BCSIR), Dhaka, 1205, Bangladesh; c Bangladesh Council of Scientific and Industrial Research (BCSIR), Dhaka 1205, Bangladesh; d Department of Chemistry, University of Dhaka, Dhaka 1000, Bangladesh; e Noakhali Science and Technology University, Noakhali, 3814, Bangladesh

**Keywords:** Photocatalytic activity, Heterojunction, Synergistic effect, Valance band offset, Conduction band offset

## Abstract

ZnO–Cu_2_O composites were made as photocatalysts in a range of different amounts using an easy, cheap, and environment-friendly coprecipitation method due to their superior visible light activity to remove pollutants from the surrounding atmosphere. X-ray diffraction and Fourier transform infrared spectroscopy (FT-IR) have demonstrated that ZnO–Cu_2_O catalysts are made of highly pure hexagonal ZnO and cubic Cu_2_O. X-ray photoelectron spectroscopy has confirmed that there is a substantial interaction between the two phases of the resultant catalyst. The optical characterizations of the synthesized ZnO–Cu_2_O composite were done via UV–vis reflectance spectroscopy. Due to the doping on ZnO, the absorption range of the ZnO–Cu_2_O catalyst is shifted from the ultraviolet to the visible region due to diffuse reflection. The degradation efficiency is affected by the Ratio of ZnO: Cu_2_O and ZnO–Cu_2_O composite with a proportion of 90:10 exhibited the most prominent photocatalytic activity on Acid Red 114, with a pseudo-first-order rate constant of 0.05032 min^−1^ that was 6 and 11 times greater than those of ZnO and Cu_2_O, respectively. The maximum degradation efficiency is 97 %. The enhanced photocatalytic activity of the composite is caused by the synergistic interaction of ZnO and Cu_2_O, which improves visible light absorption by lowering band gap energy and decreasing the rate at which the electron-hole pairs recombine. The scavenging experiment confirmed that hydroxyl radical was the dominant species for the photodegradation of Acid Red 114. Notably, the recycling test demonstrated the ZnO–Cu_2_O photocatalyst was highly stable and recyclable. These results suggest that the ZnO–Cu_2_O mix might be able to clean up environmental pollutants when it meets visible light.

## Graphical abstract

**Figure:**
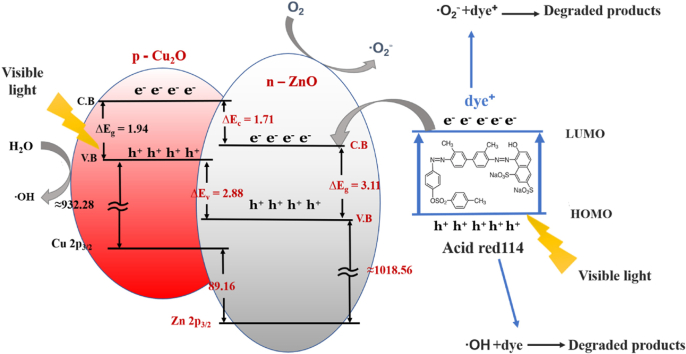

## 1 Introduction

Nowadays, photocatalysis is a viable alternative for purifying wastewater and confirmed to be a technically feasible cleanup process [1]. Industrial effluent is toxic, nonbiodegradable, and carcinogenic, making it dangerous to the environment and humans. Organic dye derivatives like acid red 114 are used in yarn dyeing and printing in textile and other industries [2,3]. Besides, the toxicity of Acid Red 114 causes severe health and environmental problems [2]. Due to their synthetic origin, toxicity, and carcinogenic or destructive nature, these dyes must be decolorized before discharge to protect humans and the environment. Organic dyes can be removed from wastewater via coagulation, adsorption, and redox methods [3]. Due to increased industrialization, environmental contamination is a global concern. This chemical industry growth discharges significant volumes of organic dangerous substances into the environment [4]. The issue is now being resolved using physical adsorption [5], photocatalysis [6], chemical oxidation [7], and biodegradation [8]. In the meantime, photocatalysis can degrade and mineralize contaminants that are challenging to completely remove from wastewater via conventional physical adsorption [9] due to its environmentally favorable nature and high efficiency. Therefore, it can be deduced that the integration of photocatalysis and adsorption processes will aid in the elimination of antibiotics [10]. The photocatalytic process experiences enhanced removal efficiency due to the expedited reaction between the pollutants and the reactive species adsorbed on the catalyst surface, which is facilitated by the high adsorption capacity of the catalyst during degradation [11].

Cuprous oxide (Cu_2_O) is a highly efficient p-type semiconductor material with a small bandgap of 2.17 eV. It has promising potential for use in many applications such as photocatalysis and photoelectric conversion [12]. For example, the n-type semiconductor ZnO, which has outstanding optical and electrical characteristics, has been identified as a viable option for creating heterostructures with other semiconductors like TiO_2_, CuO, and Cu_2_O [13]. By utilizing the suitable band alignment of Cu_2_O and ZnO, the recombination of photo-generated carriers may be effectively inhibited, leading to improved photocatalytic performance.

In this paper, ZnO has been coupled with a Cu_2_O semiconductor to demonstrate the following properties: (i) A narrow band gap is produced, which enhances visible light harvesting while ignoring UV light [14]. (ii) The formation of a p-n heterojunction reduces the rate of e^−^/h^+^ recombination and [15] (iii) increases reusability without significant efficiency loss. Cu_2_O–ZnO-GO nanocomposite was synthesized to explore the photocatalytic activity of the composite to degrade methylene blue [16]. Various forms of ZnO–Cu_2_O were constructed as well as investigated for photocatalytic activity; however, the catalytic activity was not investigated under visible light. Using the electrodeposition technique, ZnO–Cu_2_O was grown on fluorine-doped tin oxide and evaluated for electrical properties. Graphene-ZnO-Cu_2_O electrodes were built and assessed for CO_2_ electroreduction in the NaHCO_3_ electrolyte [17]. The estimated band gap energy values of ZnO–Cu_2_O were found to be 3.11 and 1.94 eV whereas Yuanyuan et al. found band gap energies of 2.82 eV and 2.62 eV for ZnO@CuO-1 and ZnO@CuO-2 core–shell heterojunction NRAs composites [18]. The maximal degradation of ZnO–Cu_2_O was found 97 % in 90 min whereas Maria et al. showed degradation of 96 % and 98 % in 120 min by BiVO_4_/Cu_2_O/G5 catalyst [19]. The preparation of Z-scheme ZnO/Cu_2_O heterojunction photocatalysts with a large specific surface area through facile liquid phase reduction method [20] but our ZnO–Cu_2_O composites with different ratios have been prepared successfully by simple precipitation and calcination methods.

Herein, we report the preparation of ZnO/Cu_2_O heterojunction photocatalysts with a specific surface area through precipitation and calcination methods. Due to formation of heterojunction, the prepared composite has been shown 98 % photocatalytic activity under the visible light which is most important part in this article. Besides, with the help of the band gap energy of the composite valence band offset (VBO) and conduction band offset (CBO) have been evaluated by XPS valence band spectra which is derived for thin film of ZnO–Cu_2_O system but here it is derived first time for powder samples to the best of our knowledge. In addition, the composite is comparatively sturdy, inexpensive, and simple to fabricate, and it is safe and non-toxic. The micromorphology and photocatalytic properties of the prepared samples are analyzed by FT-IR, XRD, XPS, UV–vis, SEM, BET and other characterization methods. Furthermore, the degradation intermediates and possible degradation paths were proposed, and the mechanism of enhancing photocatalysis performance was explored.

## 2 Experimental section

### 2.1 Materials

Copper (II) sulfate pentahydrate (CuSO_4_·5H_2_O) (99.0–100.5 %) and Potassium sodium tartrate tetrahydrate (C_4_H_4_KNaO_6_·4H_2_O, 99.0–102.0 %) were bought from Merck Germany. Commercial ZnO was obtained from Sigma-Aldrich, USA. Dextrose (C_6_H_12_O_6_) (extra pure) was purchased from Sisco Research Laboratories Pvt. Ltd. Analytical grade reagents of NaOH (98 %, Labo) and Acid Red114 (C_37_H_28_N_4_Na_2_O_10_S_3_) were used. Throughout the experiment, deionized water was used for washing and solution purification.

### 2.2 Methods

By using a precipitation method, the ZnO–Cu_2_O composite was developed. Firstly, CuSO_4_ was dissolved in water, and then 0.50 M NaOH solution was added to this solution with constant stirring until precipitation occurred. After that, Fehling solution-2 having sodium potassium tartrate was added followed by the addition of NaOH and 0.20 M glucose solution with constant stirring at 60 °C temperature to prepare pure Cu_2_O. Various amounts of ZnO and synthesized Cu_2_O; 1.8 gm & 0.2 gm, 1.5 gm & 0.5 gm, 1.0 gm & 1.0 gm, and 0.5 gm & 1.5 gm were mixed and stirred for half an hour to form a dark yellow precipitate. The precipitate was dried in an oven at 60 °C for 8 h after being repeatedly washed with deionized water. Finally, ZnO–Cu_2_O composites were synthesized with a different ratio of ZnO to Cu_2_O such as 90:10, 75:25, 50:50, and 25:75 where CuSO_4_ Concentration was 0.0475 M, 0.118 M, 0.238 M, and 0.356 M, respectively.

### 2.3 Characterization

A scanning electron microscope (JEOL, Ltd. JSM1-7600F) was used to examine the microstructure and morphological analysis of each sample. The elemental composition was examined using energy-dispersive X-ray analysis. The crystallinity as well as crystal phase of the samples were analyzed by X-ray technique, which was collected through Smart Lab SE, Multipurpose X-ray Diffractometer (XRD) with Nickel K_β_ filter and energy source copper tube 2.00 KW (40.00KV × 50.00 mA). In the experiment copper (Cu) kα radiation (λ = 1.5406 Å) where used as a standard operation mode, step size 0.01° at a scanning rate of 5°/min from two theta (2θ) range 05–80°. The diffractogram was analyzed by Smart Lab studio II software, Rigaku Corporation, Tokyo, Japan as well as phase also identified by PDF4+ ICDD data.

An X-ray photoelectron spectroscope was used to gather the elemental composition of the surface**.** The powdered samples were dispersed into ethanol followed by drop casting onto 1 × 1cm cut slide glass which was placed into the spectrometer to be examined. The XPS spectra were captured on a Thermo Fisher Scientific XPS spectrometer (7 × 10^−7^ mbar pressure) fitted with an Al kα anode (1486.68 eV). High-resolution scans of elemental lines were gathered at 200 eV pass energy for survey scans and 50 eV pass energy for narrow scans of the Hemispherical Capacitor Analyzer which yields a FWHM of the Ag 3d_5/2_ line of less than 1eV and intensity above a linear background from BE 365 eV–371 eV using 1 eV background averaging. An additional spectrum was acquired at a low pass energy (3 eV) to demonstrate an ultimate energy resolution of 0.5 eV. The standard peak positions for Cu2p_3/2_, Ag3d_5/2_, and Au4f_7/2_ were used to calibrate the spectrometer's energy scale. The peak positions usually were within 50 meV of the standard peak energies. The elemental lines binding energies had a charge of 284.8 eV when compared to the adventitious hydrocarbons C1s line.

High resolution spectra (narrow scans) were captured from the Si2p, Ca2p, O1s and C1s regions. Data were analyzed using Avantage1 software and curve fitting and deconvoluted data were obtained from the same software.

Fourier transform infrared spectrometer was used to analyze the functional groups and bonds present in the samples. A UV–visible spectrophotometer operating in the 200–800 nm wavelength range was used to observe the optical absorption spectra (Shimadzu UV-1900, Japan).

### 2.4 Photocatalytic activity

The photocatalytic activity of the ZnO–Cu_2_O composite for AR114 photodegradation in water was investigated. The dye solutions were initially made by dissolving the required amounts of dye in deionized water. As a reactor, a 100 mL glass beaker was used. A specific quantity of catalyst was taken in 20 mL of deionized water in the reactor and was sonicated for 30 min by using an ultrasonicator. Before the photocatalytic experiment began, a certain amount of dye solution was added to the sonicated suspension and stirred continuously for 60 min in the dark to make sure that the dye would adsorb and desorb evenly on the catalyst. After that, the reaction combination was positioned inside the photoreactor and exposed to visible light while being continuously stirred magnetically [21]. About 4 mL of aliquot of the suspension was taken out at every certain irradiation interval and centrifuged to separate the catalysts from the liquid. The absorbance values for the AR114 solution were measured at the *λ*_max_ = 520 nm of the dye with a UV–vis spectrophotometer [22]. The amount of dye that remains in the reaction mixture can be estimated by measuring the intensity of the dye absorbance maximum, which in turn provides an estimate of how much of the dye has been degraded. The percent efficiency has been calculated using the following formula: Percentage efficiency = $\frac{C_{o}-C}{C_{o}}$ × 100; Where, $C_{o}$ and C are the concentrations of AR114 solution at time zero and anytime of photodegradation, respectively. Ambient conditions were used for all the experiments. Five consecutive cycles of additional experiments were conducted to confirm the stability and reusability of the photocatalysts. Ethanol was used as a quenching agent in the investigation of reactive oxygen species (ROS) production. The Langmuir Hinshelwood (L-H) model has been applied to quantitatively assess the kinetics of AR114 dye degradation. This model can be applied to both gas phase and liquid phase photocatalytic reactions [22]. The linear form of the L-H equation for the pseudo 1st order kinetics can be expressed as, ln (C/C0) = -kt; where C denotes the concentration AR114 solution being degraded (gL^−1^), C0 is the primary concentration of AR114, k is the pseudo first order rate constant (min^−1^) and t is photocatalytic time (min). The linear plots of ln (C/C0) vs. irradiation time yield the reaction rate constant values, and these plots strongly correlate (R2 > 0.99) with pseudo-first-order reaction kinetics.

## 3 Results and discussion

### 3.1 Scanning electron microscopy (SEM) analysis

**Fig. 1:**
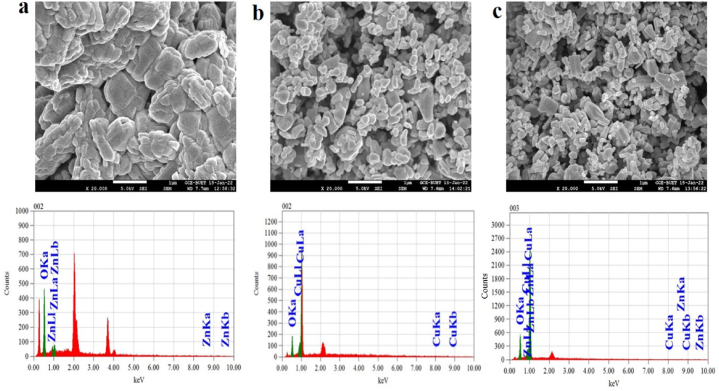
Scanning electron microscopy (SEM) images and Energy-dispersive X-ray spectroscopy (EDS) of (a) ZnO, (b) Cu_2_O, and (c) ZnO–Cu_2_O composite.

SEM was used to examine the microstructure and morphology of ZnO, ZnO–Cu_2_O, and Cu_2_O, as indicated in Fig. 1(a–c), respectively. It was clear from the SEM image that ZnO had an agglomerated morphology with uneven and nonuniform particles [23]. The prepared Cu_2_O has comparatively homogeneous spherical particles. Due to the inclusion of Cu_2_O to ZnO, the composite particles are homogeneous, uniform, and well-ordered.

The elemental composition of the samples was investigated via EDS analysis as shown in Fig. 1a-c. In the EDX spectrum of ZnO, peaks assigned to Zn and O are found. In composite, On the ZnO–Cu_2_O surface, solely the elements of O, Zn, and Cu are visible that Cu and O are present in produced Cu_2_O. Besides, no impurity peaks are detected in the composite, which further confirms that the synthesized ZnO–Cu_2_O is chemically pure [24,25].

### 3.2 X-ray diffractometry analysis

**Fig. 2:**
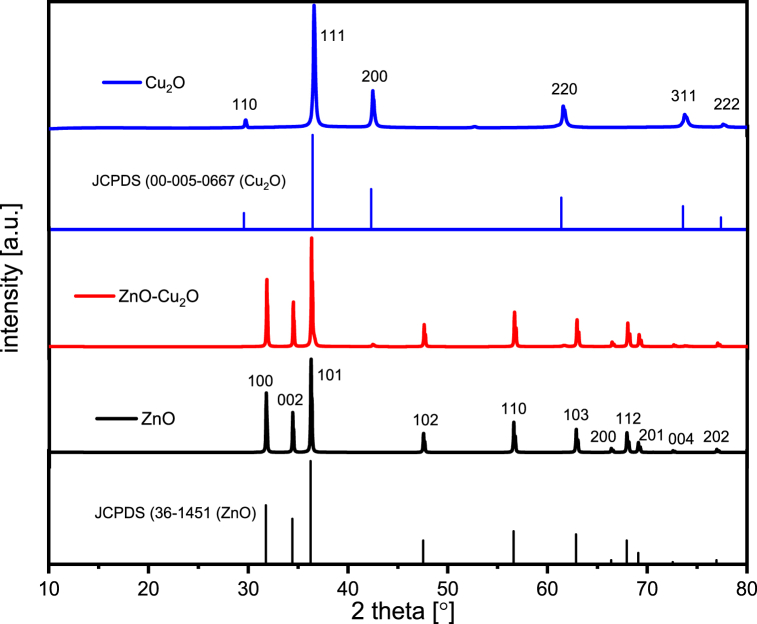
X-ray diffraction (XRD) patterns of JCPDS (36–1451) ZnO, ZnO, ZnO–Cu_2_O composite, JCPDS (00-005-0667) Cu_2_O and Cu_2_O at room temperature.

The crystallinity and crystal phase of the samples were investigated by using XRD measurements. Fig. 2 displays the XRD patterns of ZnO, ZnO–Cu_2_O and Cu_2_O respectively. The ZnO sample shows a series of diffraction peaks at 2θ values of 31.768°, 34.422°, 36.256°, 47.534°, 56.591°, 62.856°, 66.372°, 67.939°, 69.083°,72.564°, 76.944° which correspond to (100), (002), (101), (102), (110), (103), (200), (112), (201), (004) and (202) planes of the hexagonal wurtzite structure of ZnO that belongs to spatial space group *P*6_3_*mc* (186), with lattice parameters a = b = 0.3249 nm and c = 0.5206 nm according to °the diffraction card JCPDS 36–1451. Similarly, the diffraction peaks of the Cu_2_O sample obtained at the 2θ values of 29.700°, 36.590°, 42.484°, 61.567° and 73.74446° indexed as (110), (111), (200), (220) and (311) planes, respectively, are assigned to the cubic crystal phase of Cu_2_O with spatial group Pn-3m (224) 28 and lattice parameter a = b = c = 0.42696 nm which agrees with the diffraction card JCPDS 00-05-0667. The XRD characteristic reflections of ZnO in the ZnO–Cu_2_O composite are obvious, while the reflections of Cu_2_O are very weak. Because the lower amount of Cu_2_O in the composite is less than that of ZnO. The diffraction patterns of the composite show only the peaks of Cu_2_O and ZnO, suggesting that there are no other contaminants in the manufactured composite. In composite, there is little displacement between (103) and (220) reflections, which indicates the ZnO and Cu_2_O lattice interaction (12). The formation of p-n heterojunction is the result of lattice interaction, which is responsible for the enhancement of the photocatalytic activity of the ZnO–Cu_2_O composite under visible light [[26], [27], [28]].

The Debye-Scherrer method is a well-known technique for determining crystallite size by using peak broadening analysis. The average crystallite size is calculated using the Debye-Scherrer equation [29] in the following way by utilizing Equation (1) and employing the full width at half maximum (FWHM) value of the highest peak.

(1) $$D_{D-s}=\frac{k\;\lambda }{\beta_{D}\;\cos\;\theta }$$

In the given context, the symbol λ denotes the wavelength of Cu-Kα radiation (λ = 0.154060 nm). The symbol D represents the crystallite size, while the k stands for the shape factor, with a value of k = 0.9. Furthermore, $\beta_{D}$ is used to represent the integral breadth of the most intense peak, also known as the FWHM. Moreover, θ is utilized to represent the diffraction angle.

Rearranging Eq. (1) will result in the expression for the integral breadth equation (2).

(2) $$\beta_{D}=\frac{k\;\lambda }{D_{D-s}\;\cos\;\theta }$$

**Fig. 3:**
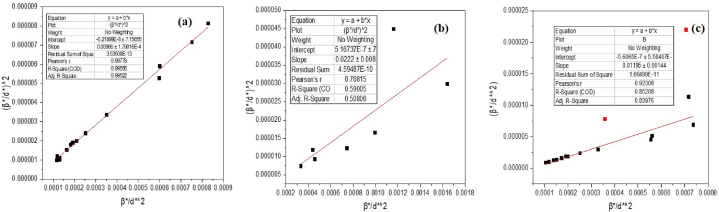
Halder-Wagner plot of (a) ZnO (b) Cu_2_O and (c) ZnO–Cu_2_O composite.

The H–W plot presented an adjusted equation (3) that incorporated the integral width, β*, of the reciprocal lattice point and the interplanar distance, d* (Fig. 3 a, b, c and Table 1). We used the Halder and Wagner approach for this analysis.

(3) $${\left(\frac{\beta^{*}}{b^{*}}\right)}^{2}=\frac{K\beta^{*}}{D_{H-W}{\left(d^{*}\right)}^{2\;}\;}+{\left(2\varepsilon_{H-W}\right)}^{2\;}$$

(4) $$\text{Where},\beta^{*}=\frac{\beta \;\cos\;\theta }{\lambda }$$

(5) $$d^{*}=\frac{2\;\sin\;\theta }{\lambda }$$

By substituting the values of β* (equation (4)) and d* (equation (5)) into Equation (6),

(6) $${\left(\frac{\beta \;\cos\;\theta }{\sin\;\theta }\right)}^{2}=\frac{K\lambda }{D_{H-W}\;}\;\frac{\beta \;\cos\;\theta }{\sin^{2}\;\theta }+16{Ɛ}_{H-W}^{2}$$

**Table 1:** Debye-Scherrer (D-S), and Halder and Wagner (H–W) plot of the ZnO, ZnO–Cu_2_O composite, and Cu_2_O crystallite size.

Samples name	Crystallite Size (nm)
ZnO using Debye-Scherrer	96.78
ZnO using Halder and Wagner (H–W) method	103.09
Cu_2_O using Debye-Scherrer	37.78
Cu_2_O using Halder and Wagner (H–W) method	45.05
Composite ZnO–Cu2O using Debye-Scherrer	85.79
Composite ZnO–Cu2O using Halder and Wagner (H–W) method	83.68

Rearranging Equation (6), We found equation (7).

(7) $${\left(\frac{\beta }{\tan\;\theta }\right)}^{2}=\frac{K\lambda }{D_{H-W}\;}\;\frac{\beta \;\cos\;\theta }{\sin^{2}\;\theta }+16{Ɛ}_{H-W}^{2}$$

Equation (6) has a resemblance to the standard linear equation where the slope is denoted as Kλ/D_(H_–_W)_ and the intercept is represented as 16Ɛ_H-W_^2^. The Halder-Wagner approach involves plotting the equation y (β/tanθ)^2^ vs x (βcosθ/sin^2^θ), as seen in Fig. 3. The data has been subjected to linear regression analysis, resulting in the determination of the crystallite size [30]. The straight lines obtained by applying the H–W method provide a satisfactory match, as seen by the correlation coefficient values of R^2^, which are ZnO, Cu_2_O and composite (ZnO–Cu_2_O) are 0.99522, 0.50806, and 0.83976, respectively.

### 3.3 Spectroscopic analysis

**Fig. 4:**
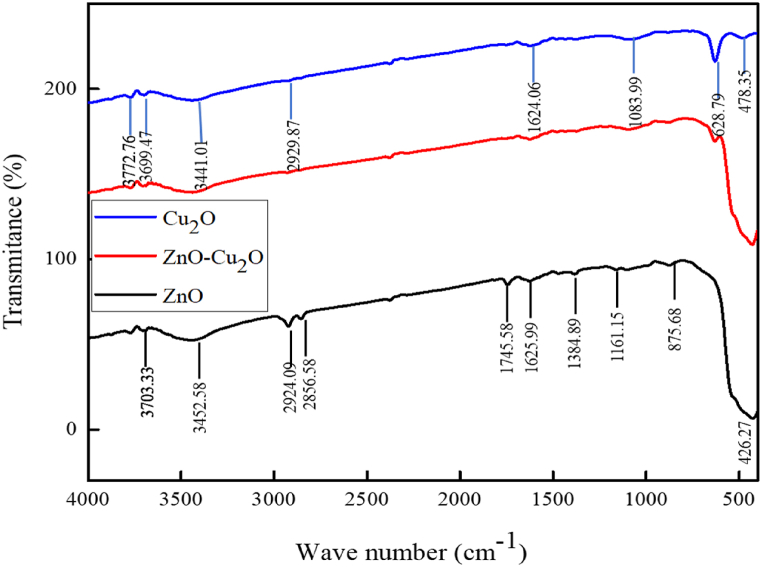
Fourier transform infrared (FT–IR) spectra of ZnO, ZnO–Cu_2_O composite and Cu_2_O.

To investigate the chemical functionality, FT-IR experiments were performed for all the samples [31,32]. The FT-IR of the ZnO, ZnO–Cu_2_O and Cu_2_O is depicted in Fig. 4. The stretching bands observed at ∼3450 cm^−1^ and ∼1480 cm^−1^ are assigned to the O–H due to moisture content for all samples. A strong peak at ∼430 cm^−1^ corresponds to the Zn–O bond. The sharp peak at ∼630 cm^−1^ which assigns to the stretching vibration band for Cu–O bond mainly originated from Cu_2_O while the sharp peak at ∼880 cm^−1^ can be correlated with vibrational frequencies due to the Cu incorporated into the ZnO lattice. The ∼2930 cm^−1^ is attributed to the C–H stretching mode. Meanwhile, the bands at ∼1385 cm^−1^ and ∼1160 cm^−1^ are related to the C–O stretching. No peaks associated with CuO, which might appear at ∼588 cm^−1^, ∼534 cm^−1^, and ∼480 cm^−1^, indicate the purity of the produced composite [33,34]. Because of the strong chemical and physical interactions between ZnO and Cu_2_O, the ZnO–Cu_2_O composite retains most of the characteristic peaks of both ZnO and Cu_2_O, with only slight changes in peak positions.

### 3.4 XPS analysis

**Fig. 5:**
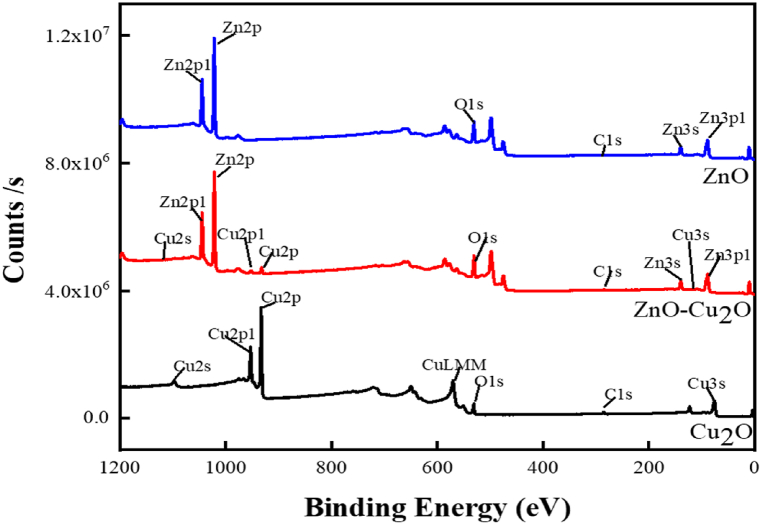
X-ray photoelectron spectroscopy (XPS) survey spectra of ZnO, ZnO–Cu_2_O composite and Cu_2_O.

**Fig. 6:**
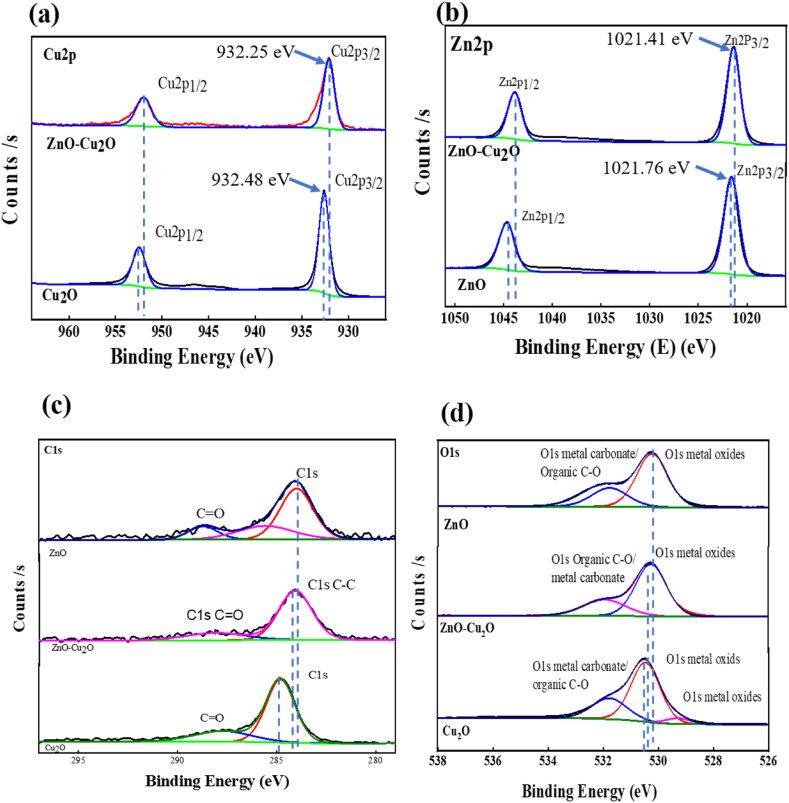
High-resolution X-ray photoelectron spectroscopy (XPS) spectra of ZnO, ZnO–Cu_2_O composite and Cu_2_O (a) Cu2p, (b)Zn2p, (c) C1s and (d) O1s.

X-ray photoelectron spectroscopy analysis was performed to determine the chemical states and composition of ZnO further precisely, ZnO–Cu_2_O, and Cu_2_O. The survey spectra of ZnO, ZnO–Cu_2_O, and Cu_2_O particles are depicted in Fig. 5. Cu2p, Zn2p, O1s, and C1s XPS high resolution spectra of the samples are displayed in Fig. 6a–d. Furthermore, because of the residual Cu(II) oxide in Cu(I)oxide, a weak satellite at 945 eV is detected, but there is no discernible satellite peak in metallic Cu which can be seen in Fig. 6a. The binding energies of Cu2P_3/2_ and Cu2P_1/2_ are responsible for the pure Cu_2_O peaks at 932.48 and 952.48 eV, which aligns with the Cu(I) species. Satellites with high-intensity shake-up and binding energies 20 eV more than [35]. The shake-up process is also responsible for the Cu 2p_1/2_ and 2p_3/2_ peaks being noticeably wider in the case of CuO. The absence of well-detectable shake-up satellites in the Cu2p spectra rules out the presence of CuO and shows that copper(I) oxide (Cu_2_O) is the main species. In Fig. 6b, the characteristic peaks at 1021.41 eV and 1044.69 eV are attributed to Zn2p_3/2_ and Zn2p_1/2_ in ZnO. Zn2p has a significantly split spin-orbit component and the 23.28 eV difference between the two binding energies confirms the +2 oxidation state of Zn in ZnO and ZnO–Cu_2_O composite [36]. The percentage of Zn2p_1/2_ and Zn2p_3/2_ of Zn(II) states in the ZnO is found to be 0.51 % and 1 %, respectively. No significant change in these states is observed in the ZnO–Cu_2_O indicating that the inclusion of Cu_2_O onto ZnO does not affect the Zn2p oxidation states of the ZnO.

It is observed that the Cu2p components of Cu_2_O have somewhat higher binding energies than ZnO–Cu_2_O. Consequently, Cu_2_O is more reactive in the composite under visible light to form electron-hole pairs as well as reactive oxidative species such as ^•^OH. For this reason, ZnO–Cu_2_O shows better photocatalytic activity under visible light. Fig. 6c represents the C1s XPS spectrum. The characteristic peaks at 284.78 eV and 288.55 eV in composite correspond to C–C and O–C]O which are responsible for adventitious carbon [36,37]. The characteristic peaks in ZnO are found at 284.71 eV and 289.16 eV. Besides, the C region found on the spectrum is due to the calibration of the XPS instrument. The O1s XPS spectra of pure ZnO, ZnO–Cu_2_O, and pure Cu_2_O are displayed in Fig. 6d. The characteristic O1s peak of ZnO–Cu_2_O at 530.26 eV corresponds to the Cu_2_O and ZnO lattice. The oxygen binding energy in the ZnO–Cu_2_O is greater than the oxygen binding energy ZnO and lower than Cu_2_O, demonstrating that the interaction between the lattice O and Zn has grown weaker and the reaction between the lattice O and Cu has grown stronger [37]. It is clear from this that lattice interaction is present in the composite.

### 3.5 Band gap energy determination

**Fig. 7:**
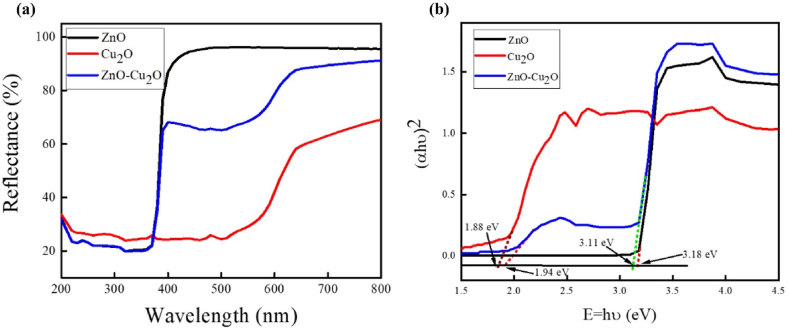
UV–vis NIR (a) Reflectance spectra of ZnO, ZnO–Cu_2_O composite and Cu_2_O and (b) Tauc plots for calculating the band gap of ZnO, ZnO–Cu_2_O composite and Cu_2_O.

Using UV–Vis NIR diffuse reflectance spectroscopy, the region of absorption of electromagnetic radiation for the nanomaterials was explored. Fig. 7a shows the UV–Vis NIR reflectance spectra of ZnO, Cu_2_O, and ZnO–Cu_2_O. It is observed that ZnO exhibits a strong adsorption band in the ultraviolet region (200–470 nm). The electron transitions from the VB to CB are primarily responsible for the absorption band edge of ZnO, which is located at 365 nm [38]. Cu_2_O reveals a single absorption band in the visible region (470–800 nm) and its absorption band edge is estimated at 540 nm. On the other hand, ZnO–Cu_2_O, exhibits two absorption bands in the UV–visible range: one at 465 nm owing to the Cu_2_O contribution and one at around 365 nm due to the ZnO contributing material. In this instance, the p-n heterojunction exhibits its characteristic behavior of shifting the absorption band of Cu_2_O to a lower wavelength. The absorption edge is associated with a substantial red shift to 465 nm in the interbond transition. The d–d transition of copper ions explains for the rise in optical absorption. It lengthens the carrier lifetime rather than increasing the quantity of photogenerated carriers, which enlarges the photocatalytic activity of the ZnO–Cu_2_O composite upon visible light irradiation [39].

To further study the optical properties of the composites, the band gap energy of the samples was estimated using the Kubelka Munk method. Fig. 7b represents the Tauc plot of the ZnO, Cu_2_O and ZnO–Cu_2_O. The relation between the absorption coefficient and the band gap energy of the materials is given in equation (8),

(8) $$\alpha h\nu =A{\left(h\nu -\text{Eg}\right)}^{n}$$

where α represents the absorption coefficient (Kubelka Munk function), h is Planck's constant, A is the absorption intensity and E_g_ denotes the band gap energy, for direct band gap n is taken equal to 1/2. Both ZnO and Cu_2_O are known to be direct band gap semiconductors. For semiconductors, when the minimum energy of the conduction band is directly above the highest energy of the valence band in momentum space then a direct band gap occurs. One of the desirable characteristics of a photocatalyst is its direct band gap since it can absorb light more effectively than materials with indirect band gaps [40]. The E_g_ is determined from the Tauc plot e.g. by plotting (αhν)^2^ vs hν, the band gap energies of ZnO, and Cu_2_O are determined to be 3.14, and 1.88 eV, respectively. Two different band gap energies of 1.94 and 3.11 eV are determined for the ZnO–Cu_2_O composite. The band gap of Cu_2_O in the composite is 1.94 instead of 1.88 for individual Cu_2_O, which lowers the e^−^/h^+^ recombination rate. The electron transfer process is briefly explained in the last part of the 3.9 section. The band gap energy of ZnO–Cu_2_O is lower than those of ZnO and Cu_2_O, confirming the successful incorporation of ZnO and Cu_2_O. Besides, lower band gap energy prospects the photocatalytic activity of ZnO–Cu_2_O upon visible light exposure.

### 3.6 BET analysis

**Fig. 8:**
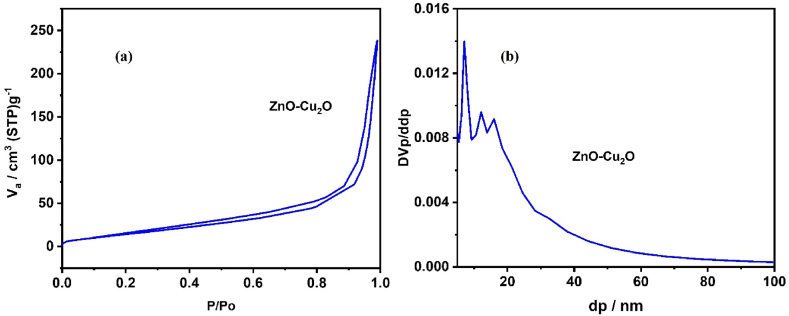
(a) N_2_-adsorption-desorption Curve of ZnO–Cu_2_O composite, and (b) BJH Curve of ZnO–Cu_2_O composite.

The N_2_ adsorption/desorption isotherm of the ZnO–Cu_2_O catalyst synthesized at room temperature are shown in Fig. 8 (a). It was possible to observe that the ZnO–Cu_2_O catalyst showed an IV type isotherm profile [41] with mesoporous structure which, in low relative pressure (P/P_0_) bands, presents relatively weak interactions between adsorption and desorption. For the higher relative pressure range, the molecular grouping is followed by pore padding, with the catalysts presenting hysteresis loop type H_3_ [42]. It was observed that the specific surface area of the ZnO–Cu_2_O catalyst was 45.12 m^2^/g. The BJH pore size distribution Curve is shown in Fig. 8 (b) which indicates that Average pore Diameter 17 nm.

### 3.7 Photocatalytic activity

**Fig. 9:**
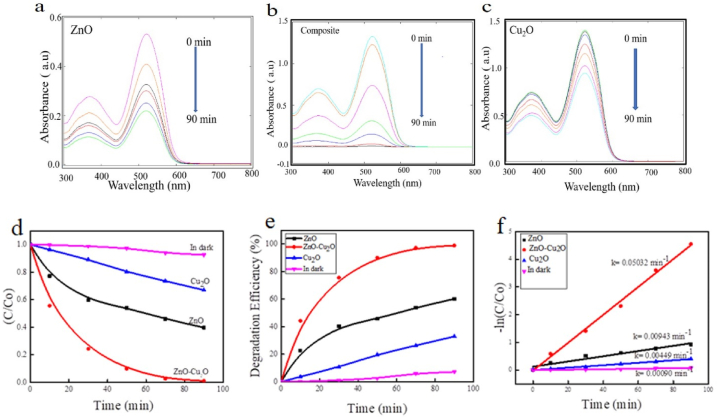
(a, b, c) comparison of photocatalytic performance through the absorption spectrum and (d) plots of C/C_O_ vs reaction time (e) plots of degradation efficiency vs reaction time (f) Pseudo-First order linear plots of ln (C/C_ο_) vs irradiation time for degradation of AR114 dye of ZnO, ZnO–Cu_2_O composite and Cu_2_O under visible light.

ZnO particles have been widely used as effective photocatalysts for organic compound degradation in the UV region [26]. To enhance the photocatalytic activity under visible light irradiation, Cu_2_O particles were incorporated into the ZnO suspension. Interestingly, his ZnO–Cu_2_O composite has shown better photocatalytic activity under visible light. The photocatalytic activities of ZnO, ZnO–Cu_2_O and Cu_2_O were investigated for AR114 degradation under visible light. The absorbance spectra from the UV–vis spectrometer exhibited a strong peak at 520 nm for AR114. Fig. 9(a–c) represents the absorption spectrum of AR114 for ZnO, ZnO–Cu_2_O and Cu_2_O. It is shown that there is a negligible change in absorption spectrum after 90 min under visible light irradiation while using ZnO and Cu_2_O separately. However, in contrast, the absorption spectrum of AR114 over ZnO–Cu_2_O composite strongly decreased after 90 min irradiation. These findings demonstrated that photodegradation of the composite was responsible for the abasement of AR114 absorption peak intensity, not the photolysis of AR114. Fig. 9d exhibits the C/C_o_ of AR114 as a function of time for all samples, where “C_o_” is the initial concentration of the dye and C is the concentration at time ‘t’. This suggests that after 90 min, there is no noticeable shift in concentration when there is no light. The concentration is drastically changed when the ZnO–Cu_2_O composite is used as a photocatalyst compared to ZnO and Cu_2_O. Fig. 9e represents the photocatalytic efficiency of samples with irradiation time [40]. When ZnO is used as a photocatalyst, about 60 % of AR114 was eliminated after 90 min exposure. Cu_2_O nanoparticles have an approximate 33 % degradation efficiency. On the contrary, the ZnO–Cu_2_O composite demonstrated noticeably improved photocatalytic efficiency towards AR114, removing approximately 100 % of AR114 after 90 min irradiation. Fig. 9f exhibits the linear correlation between ln (C/C_o_) and irradiation time for AR114 which is plausible to suggest that the photodegradation reaction appears to follow pseudo first-order kinetics following Langmuir-Hinshelwood model. The pseudo-first-order model provides data on the degradation rates kinetics of ZnO, ZnO–Cu_2_O, and Cu_2_O for AR114 photocatalytic processes. The enumerate degradation rate constants of the ZnO, ZnO–Cu_2_O, Cu_2_O and in dark condition were 9.43 × 10^−3^, 50.32 × 10^−3^, 4.49 × 10^−3^ and 0.90 × 10^−3^ min^−1^, respectively. According to these findings, the photocatalytic activity of the ZnO–Cu_2_O composite was found to be five times higher than that of ZnO nanoparticles and eleven times more than that of Cu_2_O nanoparticles [39]. This may be attributed to ZnO–Cu_2_O being an excellent photocatalyst under visible light because of the formation of p-n heterojunction and improved electrons/hole separation of the composite. Besides, from UV–Vis NIR reflectance spectroscopy, ZnO–Cu_2_O has shown superior photocatalytic activity due to small band gap energy in the visible range.

**Fig. 10:**
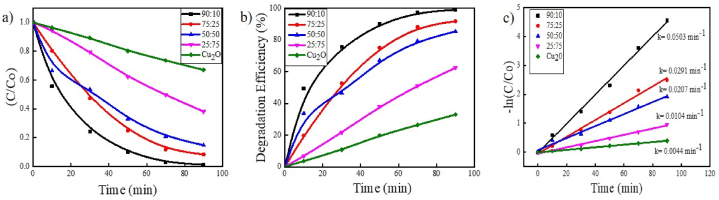
(a) Effect of different ZnO contents on ZnO–Cu_2_O composite (b) photocatalytic efficiency of different ZnO contents on ZnO–Cu_2_O composite (c) pseudo-first order linear plots of ln (C/C_ο_) vs irradiation time for degradation of AR114 dye (Composite = 0.2 g, [AR114]_ο_ = 5 × 10^−5^ M, pH = 5.5).

A variety of ZnO–Cu_2_O samples that had different ZnO and Cu_2_O proportions were generated, and their photocatalytic degradation properties were investigated, to reveal the impact of ZnO content more thoroughly on the photocatalytic performance of the ZnO–Cu_2_O photocatalyst. Fig. 10a represents the C/C_o_ vs time plot, Fig. 10b shows the photocatalytic efficiency plot and Fig. 10c exhibits kinetic studies for different ZnO content on the ZnO–Cu_2_O composite. The prepared Cu_2_O showed very poor photocatalytic activity, approximately 32 % for AR114 degradation after 90 min irradiation under visible light. The photocatalytic efficiency of Cu_2_O is significantly increased after ZnO nanoparticles are added to the composite [25]. When the ZnO/Cu_2_O ratio was 90:10, the dye degradation efficiency increased to a maximum value of about 97 % which is higher than the ZnO particle. The enlarged photocatalytic performance of ZnO–Cu_2_O reveals that there is a synergistic effect and lattice interaction between ZnO and Cu_2_O. Formation of p-n heterojunction is the result of lattice interaction which is also confirmed by XRD and XPS. To further assess the photocatalytic activity, the ZnO–Cu_2_O composite with a weight ratio of 90:10 was determined to be the ideal one.

**Fig. 11:**
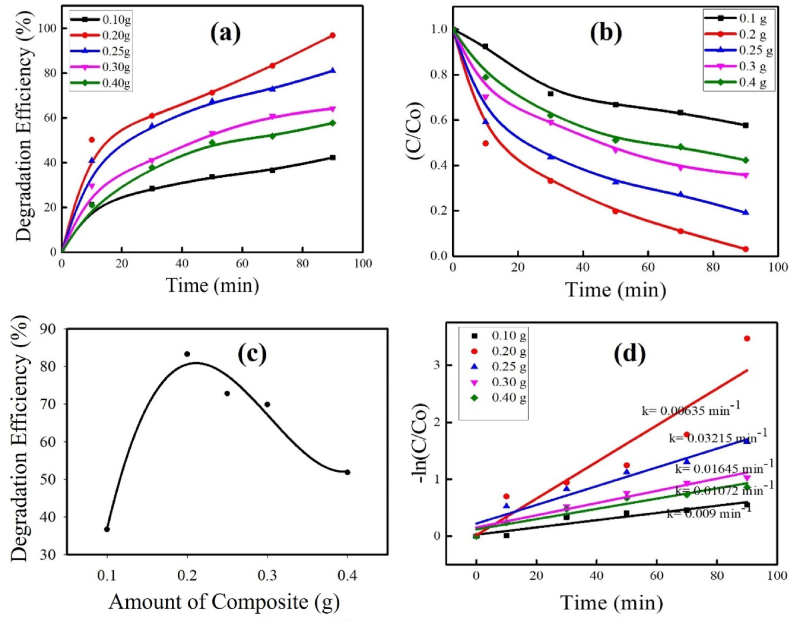
Plots for (a) different amount of composite on photocatalytic efficiency (b) C/C_o_ vs reaction time of different ZnO contents on ZnO–Cu_2_O composite (c) degradation efficiency vs amount of composite (d) pseudo-first order linear plots of ln (C/C_ο_) vs irradiation time for degradation of AR114 dye ([AR114]_ο_ = 5 × 10^−5^ M, pH = 5.30 ± 0.5).

The quantity of photocatalysts is one of the key factors influencing how well organic pollutants degrade. To investigate the optimum amount of composite, several photocatalytic degradation experiments were executed by altering the amount of ZnO–Cu_2_O composite as a photocatalyst from 0.1g to 0.6 g/100 mL remaining the other parameters constant ([AR114]_0_ = 5.00 × 10^−5^ M, pH = 5.30 ± 0.5) under visible light which is depicted in Fig. 11(a–d).

**Fig. 12:**
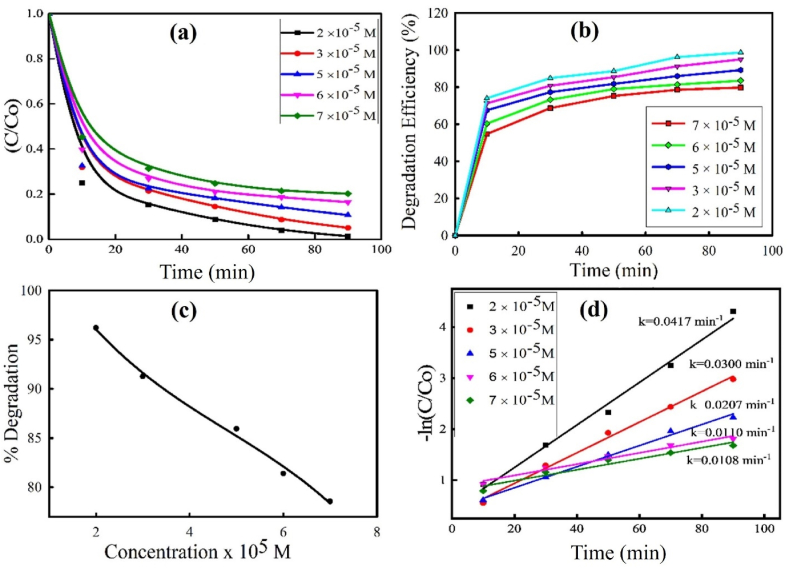
Plots for (a) C/C_O_ vs reaction time of different initial concentration of AR114 with ZnO–Cu_2_O composite (b) degradation efficiency vs reaction time (c) degradation efficiency vs different initial concentration of AR114 (d) pseudo-First order linear plots of ln (C/Cο) vs irradiation time for degradation of AR114 dye (Composite = 0.2 g, pH = 5.35 ± 0.5).

The Figures show that, as the number of composite increases from 0.1 g/100 mL, the percent degradation increases and reaches a maximum value at 0.2 g/100 mL, after that decreases with a further increase from 0.2 g/100 mL to 0.6 g/100 mL. This observation is clarified in terms of increasing the surface area, availability of more active sites on the composite surface, the absorption capacity of photons and visible light penetration into the suspension [34]. Consequently, the number of reactive ^•^OH, holes, and other reactive oxidative species (ROS) are increased which are the principal oxidizing agents in the advanced oxidation process. These facilitate the photocatalytic breakdown of the dye. When the optimum amount of composite is attained, the photodegradation efficiency decreases as the composite increases beyond 0.2 g/100 mL. The suspension becomes cloudier with increasing amounts of composite, and light cannot penetrate the bulk of the suspension. Consequently, most of the composite in bulk remains ineffective in producing the principal oxidizing agents for photodegradation, and its efficiency is diminished. This is known as light scattering and screening effects [43]. All the figures follow the same trend. Therefore, 0.20 g of composite is selected as the most suitable value for further photodegradation tests. After adjusting the other factors, such as the molar ratio and amount of composite, the photocatalytic degradation of AR114 was carried out by altering the initial concentration of AR114 to reach the appropriate concentration for maximum degradation. Fig. 12(a–d) displays the outcome of the initial concentration of dye solution by changing from 2 × 10^−5^ M to 7 × 10^−5^ M with pH = 5.35. From the figure it was observed that with an increase in the initial concentration of AR114, the percentage of photodegradation decreases [44]. The increasing dye concentration can alter light transmission, which is the cause of the declining photocatalytic degradation efficiency. Consequently, less light can penetrate the surface of the composite due to the absorption of light by the dye solution [[45], [46], [47]]. Furthermore, the total number of active sites on the composite surface may be restricted by a specific quantity of composite, and an inadequate amount of hydroxyl radicals may form. Hence, the photodegradation efficiency has decreased.

### 3.8 Recyclability

**Fig. 13:**
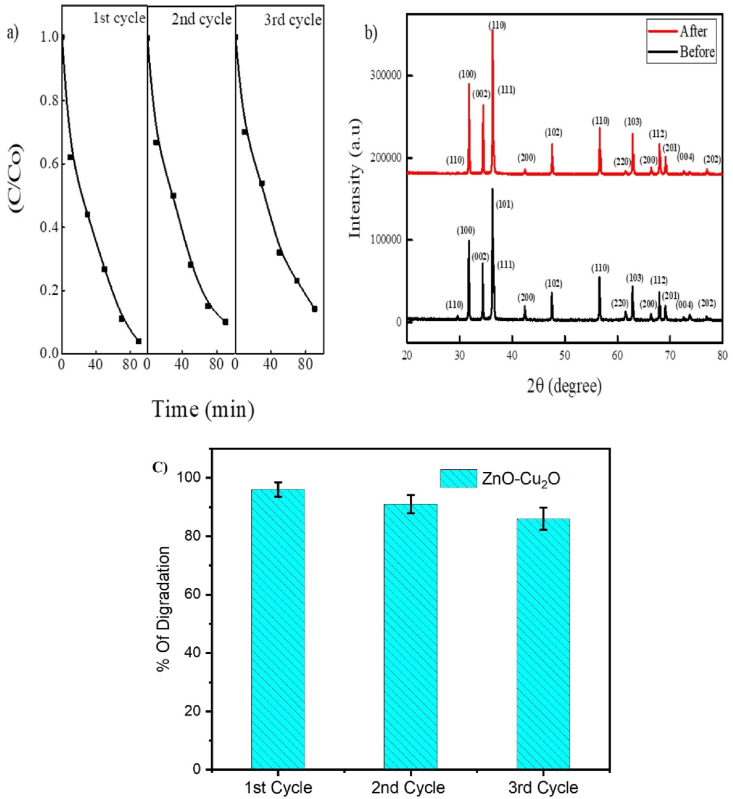
(a) Photocatalytic activity of the ZnO–Cu_2_O for acid red 114 degradation with three times of cycling uses ([AR 114] = 2 × 10^−5^ M; photocatalyst amount = 0.20 g; pH = 5.2). (b) XRD pattern of ZnO–Cu_2_O after reused for three cycles and (c) error bar represent the standard deviation of triplicate measurements for percent degradation.

Recyclability is an essential factor in considering the practical applicability of photocatalysts. To investigate photocatalytic stability, For the recyclability experiment, ZnO–Cu_2_O is used up to three times, as Fig. 13a demonstrates. After every photocatalytic cycle, the composite is removed by centrifugation, rinsed, and dried before the next cycle can be carried out. The catalyst is exposed to radiation for 90 min under visible light throughout each cycle. Every cycle shows a slight decrease in the composite's photocatalytic activity [39]. The percentage of dye removed after three cycles of photocatalysis was 86 % as compared to 96 % at the first cycle. The agglomeration of catalyst particles and subsequent weight loss throughout the centrifugation and washing procedures of the composite are responsible for the gradual decrease in degrading efficiency [25]. The XRD pattern of the ZnO–Cu_2_O composites is shown in Fig. 13b before and after three cycles of photocatalytic reactions to test how stable the composites are when exposed to light. The characteristic XRD patterns of ZnO and Cu_2_O in the composite were apparent before and after the photocatalytic recycle tests, demonstrating that ZnO–Cu_2_O composites are photostable and exhibit fewer corrosive properties. So, it was shown that ZnO–Cu_2_O was a good photocatalyst for breaking down AR114 when exposed to visible light and could be used as a photocatalyst in different situations. The error bars demonstrating the standard deviation of the triplicate measurements for percent degradation is displayed in Fig. 13 (c).

### 3.9 Active species responsible for AR114 degradation

**Fig. 14:**
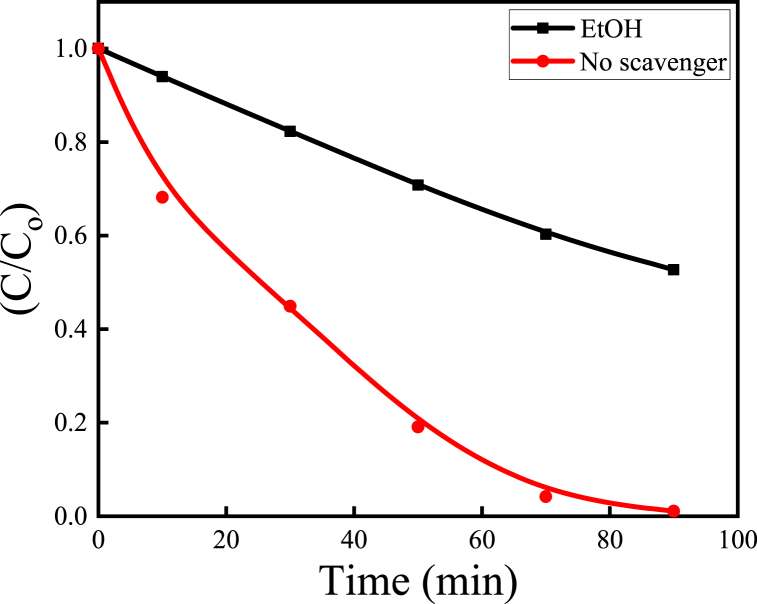
Effect of ethanol as a scavenger on degradation of AR114 in the presence of ZnO–Cu_2_O (composite = 0.2 g, [AC114] _ο_ = 2.0 × 10^−5^ M, pH = 5.30).

To investigate the predominant oxidative species responsible for AR114 degradation, comparative studies of scavenger loaded conditions have been conducted using ZnO–Cu_2_O nanocomposite under optimized conditions (composite = 0.2 g, [AR114]_o_ = 2.0 × 10^−5^ M, pH = 5.30). With the aid of suitable quenchers, the formation of conceivable oxidative intermediate species like ^•^OH, or superoxide anion radicals (^•^O_2_^−^), if they exist under photoreaction, and their role in the degradation of AR114 were investigated. In this case, ethanol (EtOH) was used in the photocatalytic reaction as the ^•^OH scavenger [44]. Fig. 14 demonstrates the C/C_o_ and the irradiation time plot of the ZnO–Cu_2_O composite after the addition of EtOH as a scavenger. The figure depicts that the photodegradation of AR114 was significantly reduced in the presence of EtOH compared to no scavenger under identical conditions evaluating that the ^•^OH was the primary oxidative species responsible for AR114 degradation [45]. From this study, it can be argued that ^•^OH play a significant factor in the degradation process.

**Fig. 15:**
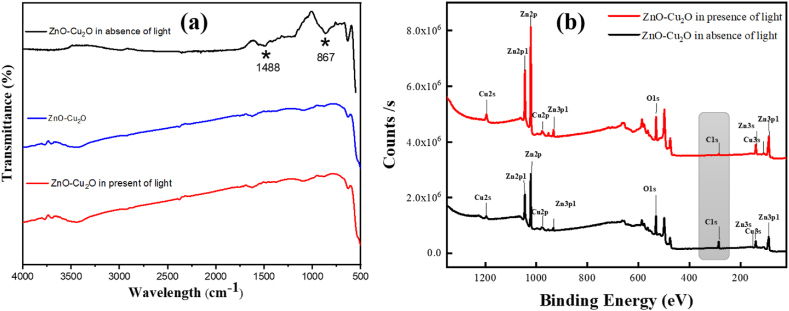
(a) FT-IR spectra and (b) XPS survey spectra of ZnO–Cu_2_O composite in presence and absence of light.

In Fig. 15a, The FT-IR spectrum of the ZnO–Cu_2_O composite displays two peaks at 867 and 1488 cm^−1^ for C]C bending, and S]O stretching modes which is absent when light irradiates into the sample. Similarly, XPS analysis reveals that when the composite is exposed to light, there is no longer a C1s peak for the ZnO–Cu_2_O composite in Fig. 15b, These data indicate that AR 114 dye fully degraded in the presence of light.

The following formula can be used to determine the valence band offset (VBO) at the heterojunction interface of the ZnO–Cu_2_O composite [46]:

(9) $${\Delta E}_{V}=\left(E_{{Cu}_{2}P}^{{Cu}_{2}O}-E_{{Zn}_{2p}}^{ZnO}\right)+\left(E_{{Cu}_{2}P}^{{Cu}_{2}O}-E_{VBM}^{{Cu}_{2}O}\right)\;-\;\left(E_{{Zn}_{2p}}^{ZnO}-E_{VBM}^{ZnO}\right)$$

where $\left(E_{{Cu}_{2}P}^{{Cu}_{2}O}-E_{VBM}^{{Cu}_{2}O}\right)$ deals with the energy separation between Cu2p as well as valence band maximum (VBM) in Cu_2_O, $\left(E_{{Zn}_{2p}}^{ZnO}-E_{VBM}^{ZnO}\right)$ is the energy separation between Zn2p and VBM in ZnO, $\left(E_{{Cu}_{2}P}^{{Cu}_{2}O}-E_{{Zn}_{2p}}^{ZnO}\right)$ is the energy difference between Cu2p and Zn2p core levels (CLs) at the interface of ZnO–Cu_2_O composite. ${\Delta E}_{V}$ is the VBO of the composite.

**Fig. 16:**
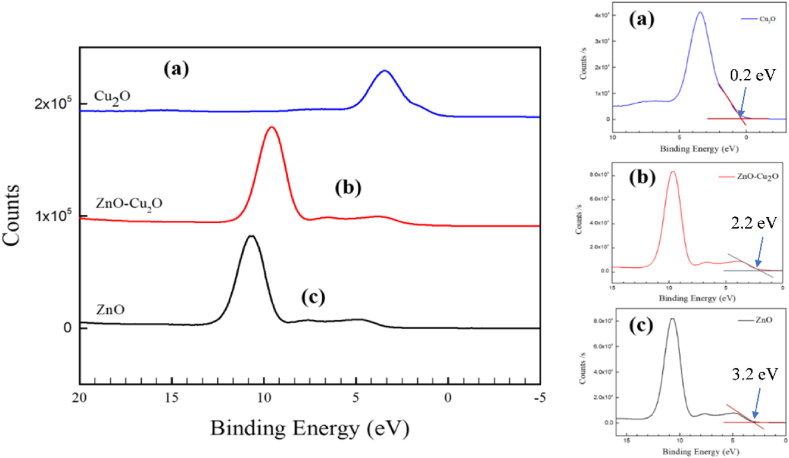
XPS valance band spectra of (a) the Cu_2_O (b) ZnO–Cu_2_O composite and (c) ZnO.

The XPS valence band spectra of Cu_2_O, ZnO, and ZnO–Cu_2_O composite are depicted in Fig. 16(a–c). The valence band maximum (VBM) value has been estimated to be 3.2, 2.2 and 0.2 eV for ZnO, ZnO–Cu_2_O and Cu_2_O. The VBM placements are determined by finding the cut point between the leading edges of the linear extrapolation from the valence band about the backdrop [47].

According to Eq. (9), the VBO of ZnO–Cu_2_O are determined to be 2.53 eV. The conduction band offset (CBO) of ZnO–Cu_2_O heterojunction can be estimated by the following equation (10).

(10) $${\Delta E}_{c}=\left(E_{g}^{ZnO}-E_{g}^{{Cu}_{2}O}-{\Delta E}_{V}\right)$$

**Fig. 17:**
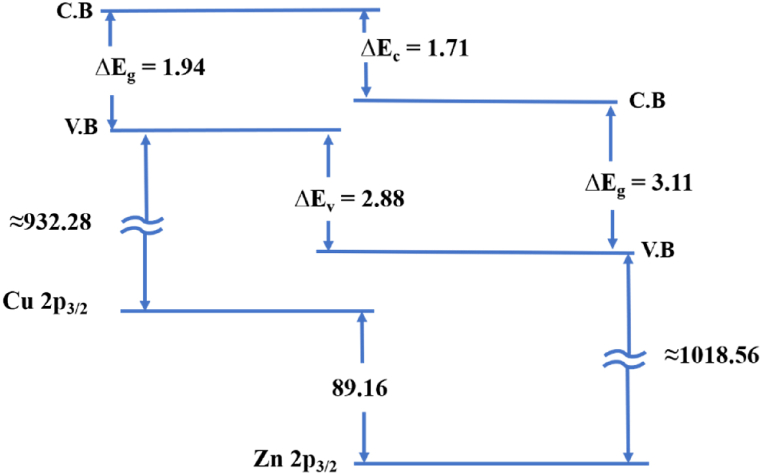
Schematic diagram of band alignment of ZnO–Cu_2_O heterojunction.

At normal temperature, the band gaps of two different band gap energies of ZnO–Cu_2_O composite are 1.94 and 3.11 eV. As a result, Cu_2_O has a higher CB level than ZnO, and the estimated CBO for ZnO–Cu_2_O are 1.36 eV. The schematic diagram of the band alignments is displayed in Fig. 17. The ZnO–Cu_2_O VBO value that we found here is 2.53 eV which differs from the value [48]. One explanation could be that various preparation techniques were used, which contributed to the Cu_2_O films.

For the fabricated heterojunction, the computed values for VBO and CBO are determined to be 2.53 eV and 1.36 eV. The comparatively large VBO value extracted here lessens the transfer of holes from Cu_2_O to ZnO [46]. It would also help to suppress light emission in the visible area. This high concentration of holes in the valance band of Cu_2_O facilitates the H_2_O to ^•^OH reaction as this reaction occurs on the Cu_2_O surface. Considering the band gap energies for ZnO and Cu_2_O, the electrons readily transfer from the CB of Cu_2_O to CB of ZnO. The high concentration of electrons in the ZnO surface increases the rate of O_2_ to ^•^O_2_^−^ reaction thus increasing the degradation rate of Acid red114 dye.

### 3.10 Proposed mechanism

**Fig. 18:**
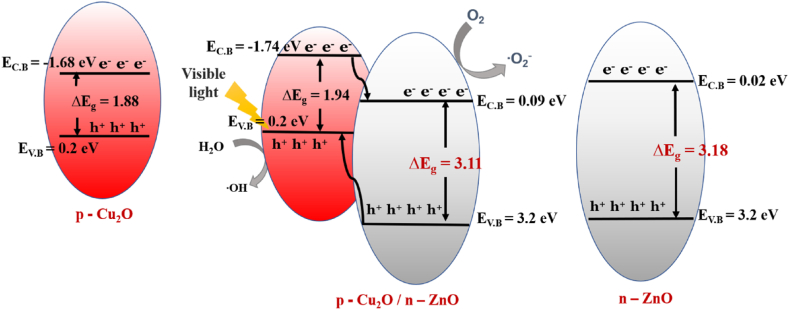
A Schematic diagram of the highly efficient photocatalytic activity for ZnO–Cu_2_O composites under visible light.

It is discovered that the ZnO–Cu_2_O composite exhibits greater photocatalytic activity compared to ZnO and Cu_2_O. The increased optical absorption is responsible for the higher photocatalytic activity and the configuration of p-n heterojunction which encourages photo-introduced electron/hole pair separation in ZnO–Cu_2_O. The mechanism of the photocatalytic reaction ZnO–Cu_2_O p-n heterojunction is schematically explained in Fig. 18.

The reaction steps are described below,

a) Light absorption by the photocatalyst results in the following reactions:

**Figure:**
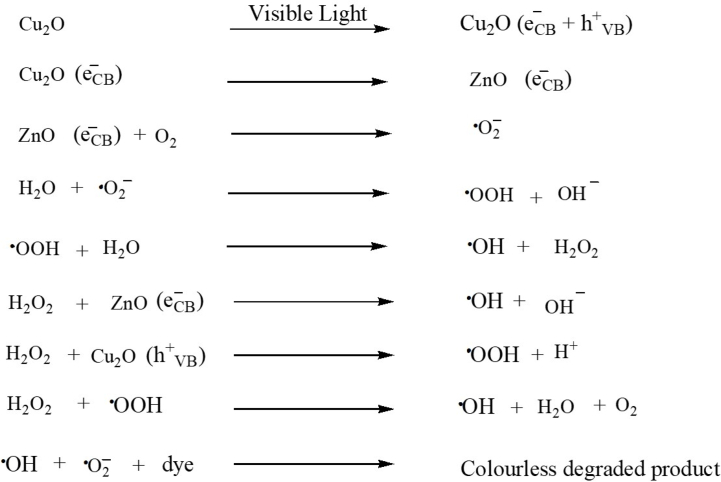

b) For dyes, the following processes result from light absorption:

**Figure:**
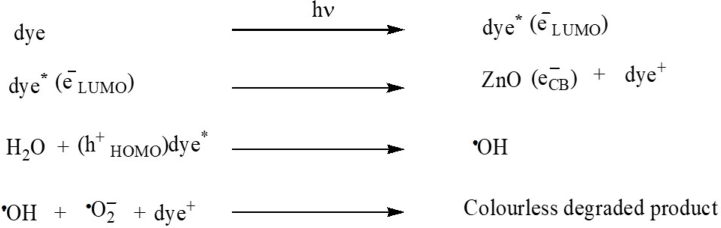

Because of its small band gap energy, Cu_2_O produces electron-hole pairs in the valance band and conduction band when the ZnO–Cu_2_O is exposed to visible light, respectively. Compared to the corresponding ZnO, the Cu_2_O conduction band is more negative. So, the photo-induced electrons get a downhill flow from CB of Cu_2_O to CB of ZnO generating an internal electric field across the interface. The process of charge pair recombination is suppressed by the induced electric field across the interface. Enough electrons and holes are produced by the charge separation phenomenon for the redox reaction, which yields enough active radicals to support photocatalytic activity [35]. The electrons at the conduction band of ZnO undergo a reaction with dissolved oxygen to give a peroxide radical anion and ^•^O_2_^−^ which in turn produces H_2_O_2_ in the presence of water. The H_2_O_2_ is then reacted with the ^•^O_2_^−^ to generate the powerful ^•^OH. Besides, the ^•^OH is oxidized by holes of VB of Cu_2_O and produces an oxidizing agent the ^•^OH. Eventually, the ^•^OH is the main oxidizing agent to convert the dye into photocatalytic degraded products. Under visible light, dyes are also excited to dye*. Therefore, photogenerated electrons of dye* are transferred from the LUMO level of dye* to the CB of ZnO. H_2_O receives a hole from the HOMO level of dye* to generate ^•^OH directly which degrades the dye into degraded products. ZnO exhibits photocatalytic activity due to the photoexcitation of dye under visible light.

## 4 Conclusion

ZnO–Cu_2_O composites with different ratios have been prepared successfully by simple precipitation and calcination methods. UV–vis near IR reflectance spectroscopy, FT-IR, XRD, XPS, and SEM were used to characterize the composite. SEM image revealed that the ZnO–Cu_2_O composite is comparatively uniform, well-ordered morphology than that of ZnO. The UV–vis absorbance of ZnO–Cu_2_O showed enhanced absorption in the visible range with two different band gaps of 1.94 and 3.11 eV and suppressed the recombination rate of e^−^/h^+^ through the formation of p-n heterojunction. As a result, with a photocatalytic rate constant of 50.32 × 10^−3^ min^−1^, the ZnO–Cu_2_O photocatalyst demonstrated superior photocatalytic efficiency for AR114 degradation compared to ZnO and Cu_2_O. Scavenger experiments suggest that the ^•^OH is the main active species during the photocatalytic degradation process. ZnO–Cu_2_O also showed relative stability throughout each cycle of recyclability without experiencing a discernible decrease in photocatalytic activity, indicating that it has a viable and appropriate use for the treatment of wastewater contaminated with dyes.

## Data availability statement

The data described in the present research are accessible from the authors who wrote it upon request.

## CRediT authorship contribution statement

**Nasrin Akter:** Writing – original draft, Visualization, Methodology, Formal analysis, Data curation. **Tanvir Ahmed:** Writing – original draft, Visualization, Methodology, Formal analysis, Data curation. **Imdadul Haque:** Writing – review & editing, Visualization, Methodology, Formal analysis, Data curation. **Md Kamal Hossain:** Visualization, Investigation, Formal analysis. **Gorungo Ray:** Visualization, Formal analysis, Data curation. **Md Mufazzal Hossain:** Writing – review & editing, Formal analysis. **Md Sagirul Islam:** Visualization, Formal analysis, Data curation. **Md Aftab Ali shaikh:** Writing – review & editing, Supervision, Investigation. **Umme sarmeen Akhtar:** Writing – review & editing, Visualization, Validation, Supervision, Software, Resources, Project administration, Methodology, Investigation, Formal analysis, Data curation, Conceptualization.

## Declaration of competing interest

The authors declare that they have no known competing financial interests or personal relationships that could have appeared to influence the work reported in this paper.
